# Supplementary material for: DrivR-Base: a feature extraction toolkit for variant effect prediction model construction
Source: Bioinformatics. 2024 Apr 11;40(4):btae197. doi: 10.1093/bioinformatics/btae197 (PMC11057939; doi:10.1093/bioinformatics/btae197)
Supplement: btae197_Supplementary_Data [file btae197_supplementary_data.pdf]

DrivR-Base Supplementary Material

Amy Francis, Colin Campbell, Tom Gaunt

March 1, 2024

1 Introduction

In this Supplementary, we present resources available within *DrivR-Base* for downloading data which is informative for predicting the functional impact of variants in the human genome. The scripts in DrivR-Base are organized into molecular feature groups (designated as FG1, FG2, etc.). Within each resource, we provide four subsections to guide the users. Firstly, we provide a brief background explaining why the data source can provide valuable insights for predicting the pathogenic status of a variant. We then provide information about the data sources and locations, and details on how to extract the data, including any useful scripts or tools for the extraction process.
Here, we provide a collection of useful scripts for efficiently aggregating feature data. Our resource compilation, presented below, focuses on the human genome, providing extraction scripts and data location indicators specifically for build GRCh38/hg38 of the human genome.

2 Software Availability

DrivR-Base source code is available at https://github.com/amyfrancis97/DrivR-Base.

3 Feature Groups

FG1: Conservation-Based Features

Background: Sequence conservation across species serves as a valuable indicator for predicting the pathogenicity of variants. DNA regions which are highly conserved across species have an expectation of being functionally significant. Conversely, there is an indication that variants in poorly conserved regions generally have more limited functional effects and are therefore better tolerated (Woodruff, 2001).

Data source: http://hgdownload.cse.ucsc.edu/goldenpath/hg38/

Script location: https://github.com/amyfrancis97/DrivR-Base/tree/main/FG1_conservation

Extraction: To incorporate conservation-based features, we download 22 datasets comprising Bismap and Umap multi-way mappability scores (Karimzadeh et al., 2018), and PhyloP and PhastCons conservation measures (Pollard et al., 2009; Siepel et al., 2005), from the UCSC Genome Browser Golden Path (Table 1 and 2).

Table 1: Conservation Features

Conservation Features	
phyloP4way	phastCons4way
phyloP7way	phastCons7way
phyloP17way	phastCons17way
phyloP20way	phastCons20way
phyloP30way	phastCons30way
phyloP100way	phastCons100way
phyloP470way	phastCons470way

Table 2: Mappability Features

Mappability Features	
k24.Bismap.MultiTrackMappability	k24.Umap.MultiTrackMappability
k36.Bismap.MultiTrackMappability	k36.Umap.MultiTrackMappability
k50.Bismap.MultiTrackMappability	k50.Umap.MultiTrackMappability
k100.Bismap.MultiTrackMappability	k100.Umap.MultiTrackMappability

We then convert the files to bedGraph format using the package: https://anaconda.org/bioconda/ucsc-bigwigtobedgraph. To align the variant file sets with the downloaded datasets, a data reformatting step is necessary to ensure compatibility. Specifically, we transform the original format of chromosome, start, end, reference allele, and alternate allele to the format of chromosome, start-1, end, reference allele, and alternate allele. Once reformatted, we query the downloaded conservation and mappability scores against the variant file using bedtools intersect. The output of this script are 20 separate BED +1 files containing the chromosome location, reference and alternate alleles, and the associated score. Please see the 'get_conservation.sh' script in the '/DrivR-Base/FG1_conservation' folder of our GitHub repository for more details

FG2: Variant Effect Predictor

Background: Ensembl's Variant Effect Predictor (VEP) (McLaren et al., 2016) is a tool that offers a diverse array of features that can be predicted for single nucleotide variants. The features used in this analysis include predicted consequences on transcripts, distances from transcripts, and amino acids that are predicted to be impacted by the variant. The consequences that are predicted by VEP and a description can be found in Table 3. Predicting effects on transcripts offers valuable insights into functional significance. For example, variants that are predicted to lead to more severe consequences, such as the introduction of premature stop codons, or frameshift variants, are more likely to be pathogenic and to play a role in disease. The predicted amino acids from VEP enable us to establish whether a variant is synonymous and whether a non-synonymous variant leads to an amino acid whose chemical properties differ substantially from the wild type. Hence, by considering the predicted amino acid changes, we gain insights into the potential functional implications of variants and their association with disease.

Table 3: Consequences of Genetic Variants

Consequence	Description
splice_acceptor_variant	Variant affects splice acceptor site
splice_donor_variant	Variant affects splice donor site
stop_gained	Gain of a stop codon
frameshift_variant	Indel causes a frameshift
stop_lost	Loss of a stop codon
start_lost	Loss of a start codon
transcript_amplification	Amplification of a transcript
feature_elongation	Elongation of a genomic feature
feature_truncation	Truncation of a genomic feature
inframe_insertion	In-frame insertion of bases
inframe_deletion	In-frame deletion of bases
missense_variant	Missense mutation
protein_altering_variant	Protein-altering variant
splice_donor_5th_base_variant	Variant at the 5th base of a splice donor site
splice_region_variant	Variant in a splice region
splice_donor_region_variant	Variant in a splice donor region
splice_polypyrimidine_tract_variant	Variant in a polypyrimidine tract
incomplete_terminal_codon_variant	Incomplete terminal codon
start_retained_variant	Start codon retained
stop_retained_variant	Stop codon retained
synonymous_variant	Synonymous mutation
coding_sequence_variant	Variant in coding sequence
mature_miRNA_variant	Variant in mature miRNA
5_prime_UTR_variant	Variant in 5' untranslated region
3_prime_UTR_variant	Variant in 3' untranslated region
non_coding_transcript_exon_variant	Variant in non-coding transcript exon
intron_variant	Variant in intron
NMD_transcript_variant	NMD (Nonsense-Mediated Decay) transcript variant
non_coding_transcript_variant	Variant in non-coding transcript
coding_transcript_variant	Variant in coding transcript
upstream_gene_variant	Variant upstream of a gene
downstream_gene_variant	Variant downstream of a gene
TFBS_ablation	Transcription factor binding site (TFBS) ablation
TFBS_amplification	Transcription factor binding site (TFBS) amplification
TF_binding_site_variant	Variant affects TF binding site
regulatory_region_ablation	Regulatory region ablation
regulatory_region_amplification	Regulatory region amplification
regulatory_region_variant	Variant affects regulatory region
intergenic_variant	Intergenic variant
sequence_variant	Generic sequence variant

Data source: https://www.ensembl.org/info/docs/tools/vep/script/vep_cache.htmlcache

Script location: https://github.com/amyfrancis97/DrivR-Base/tree/main/FG2_vep

Extraction: To utilize the Variant Effect Predictor (VEP) for variant annotation, we begin by

downloading the VEP cache to our local environment. A list of prerequisites and dependencies for cache download can be found in our DrivR-Base GitHub repository. Additional information is available in the VEP cache tutorial online. Once the VEP cache is downloaded locally, we can proceed to query and extract relevant features for specific variants. For a detailed description of the querying process, please refer to our 'get_vep.sh' script located in the '/DrivR-Base/FG2_vep' sub-directory of the repository.

We extract these features as separate queries to make the output more interpretable and, after querying features for the given variant, we reformat this output to similarly enhance useability. For consequence features, we implement a one-hot-encoding approach. Each possible consequence is represented as a distinct column. A '1' in a column indicates the prediction of a specific consequence, while '0' denotes its absence. Similarly, for amino acid features, we concatenate two one-hot-encoded vectors. The first vector corresponds to the wild-type amino acid, with each element representing one of the 20 amino acids. A '1' in the vector indicates the amino acid coded by the wild-type codon. The second vector follows the same structure but relates to the mutant amino acid. The resulting two vectors are then concatenated to form the final amino acid feature representation.

FG3: Dinucleotide Properties

Background: In our study, we use features derived from DiProDB (Friedel et al., 2009), a comprehensive database of conformational and thermodynamic dinucleotide properties. We can use this information to elucidate any conformational and thermodynamic changes that occur between wild-type and mutant genomic sequences, hence providing insight into the potential functional significance of variants on a DNA-level.

Data source: https://diprodb.fli-leibniz.de/ShowTable.php

Script location: https://github.com/amyfrancis97/DrivR-Base/tree/main/FG3_dinucleotide_properties

Extraction: For each variant, we extract values for four dinucleotide configurations in R: 1) The wild-type allele paired with the adjacent allele on the left, 2) The wild-type allele paired with the adjacent allele on the right, 3) The mutant allele paired with the adjacent allele on the left, and 4) The mutant allele paired with the adjacent allele on the right. Each configuration contains 125 dinucleotide property features, resulting in a dataframe containing 500 feature columns for each variant, with corresponding row values from the original DiProDB table. The script for this extraction process is 'dinucleotide_properties.R' and can be found in '/DrivR-Base/FG3_dinucleotide_properties'

FG4: DNA Shape Properties

Background: We use DNAShapeR (Chiu et al., 2016) to capture DNA shape properties of genomic variant sites. Specifically, this package uses a sliding pentamer window approach and Monte Carlo simulations to determine the minor groove width (MGW), helix twist (HelT), propeller twist (ProT), and roll (Roll) values for DNA sequences. Crucially, DNA shape features are well-established for their roles in protein-DNA binding specificity (Jones et al., 2003; Chiu et al., 2017; Rohs et al., 2009). Given the significance of these DNA shape features in protein-DNA interactions, it is reasonable to hypothesize that variants that impact these

shapes may have greater pathogenic potential. By incorporating these DNA shape features into our analysis, we aim to capture the impact of variants on protein-DNA binding and explore their potential association with pathogenicity.

Data source: https://www.ncbi.nlm.nih.gov/pmc/articles/PMC4824130/

Script location: https://github.com/amyfrancis97/DrivR-Base/tree/main/FG4_dna_shape

Extraction: We read variant datasets into R and extract DNA nucleotides flanking ten base pairs either side of the variant of interest. This results in a 21 base-pair DNA sequence, where base 11 denotes the variant. We then pass this sequence to the 'getShape()' function in 'DNAShapeR'. This calculates MGW, HelT, ProT, and Roll values for each of the positions in the wild-type nucleotide sequence. This enables us to establish whether variants found in regions exhibiting particular dna shape properties are more likely to lead to pathogenic effects. For detailed steps on the implementation of this process, please refer to the 'dna_shape.R' script located in the '/DrivR-Base/FG4_dna_shape' directory of the repository.

FG5: GC and CpG content

Background: The GC content, also referred to as the *isochore* structure, exhibits significant heterogeneity in the mammalian genome. While many questions still remain regarding the functional implications of these organizational events, studies have associated these structures with various genomic features that hold potential functional significance in the context of driver prediction. For example, increased bendability and B-Z transitions have been associated with elevated GC content and, these structures have been linked to open chromatin and active transcription (Vinogradov, 2003). DNA curvature has been associated with condensed chromatin state (Vinogradov, 2003). This feature group also include CpG counts and observed vs expected ratios. CpG sites are frequently enriched in gene promoters within mammalian genomes (Rozenberg et al., 2008). Hence, there is potentially a gain to be made in including these feature groups when predicting the functional impact of genomic variants.

Script location: https://github.com/amyfrancis97/DrivR-Base/tree/main/FG5_gc_CpG

Extraction: We first download the GRCh38 human genome fasta files from NCBI (Sayers et al., 2022). We then query the genome to extract nucleotide sequences surrounding the variant of interest. We repeat this for 9 different window sizes. For each of these sequences, we then calculate GC content:

$$GCcontent = \frac{Count(G+C)}{Count(A+T+G+C)} \times 100\% \tag{1}$$

Next, we calculate the observed vs expected ratio:

$$Obs/Exp(CpG) = \frac{Count(CpG)}{Count(C+G)} \times n \tag{2}$$

Where $Count(CpG)$ is the number of CG dinucleotides, and n is the nucleotide sequence length.

FG6: Kernel-Based Sequence Similarity

Background: We employ sequence-based p-spectrum kernels to capture potential disruptions in sequences flanking a single nucleotide variant (Campbell and Ying, 2011). Spectrum kernels are used to characterize the composition of k-mers within regions surrounding the SNV, both before and after the mutation occurs. The result of the spectrum kernel algorithm is the generation of two k-spectra: one representing the wild-type version of the sequence and the other representing the mutant version. These k-spectra encode the frequency and distribution of k-mers within their respective sequences. By concatenating these two k-spectra, we obtain a comprehensive representation that encapsulates the complete picture of how the sequence changes due to the SNV.

Script location: https://github.com/amyfrancis97/DrivR-Base/tree/main/FG6_kernel

Extraction: Similarly to the previous feature group, we first download the GRCh38 human genome fasta files from NCBI (Sayers et al., 2022). We then query the genome to extract nucleotide sequences surrounding the variant of interest for window sizes ranging between 2 and 10. In the 'get_kernel.py' script, we created an 'over_slice()' function which systematically generates all possible combinations of k-mers of given sizes, k, for each pair of wild type and mutant sequences using a sliding window approach. The local sequence patterns around the variants can then be calculated from these k-mers. To count the occurrence of each k-mer, we employ a mapping function:

$$\Phi_u^p(s) = |\{(v_1, v_2) : s = v_1 u v_2\}|$$

Here, u represents the sub-string k-mer of length p, v_1 denotes the wild-type sequence, v_2 refers to the mutant sequence, and s represents the sequence of interest. The mapping function quantifies the number of times the substring u appears in the sequence s, considering both the wild-type and mutant sequences. For example, let's consider the sequences "ATCGT" and "ATAGT", where the mutant sequence has replaced a cytosine residue with an adenine. If we use a k-mer size of 2, the result of the mapping function would be:

Table 4: p-spectrum kernel mapping function

**seq**	**AT**	**TC**	**CG**	**GT**	**TA**	**AG**
ATCGT (s)	1	1	1	1	0	0
ATAGT (t)	1	0	0	1	1	1

From the mapping function, we can derive a p-spectrum kernel matrix by summing the products of the corresponding row entries for the two sequences:

$$K_p(s,t) = \sum_{u \in_p} \Phi_u^p(s) \Phi_u^p(t)$$

In this equation, s corresponds to the wild-type sequence, and t corresponds to the mutant sequence. The practical procedure involves the following steps:

1) Count the number of 'AT' k-mers in the wild-type sequence (s), which results in a count of 1.
2) Count the number of 'AT' k-mers in the mutant sequence (t), which also results in a count of 1.
3) Multiply the counts obtained in steps 1 and 2 (1 * 1 = 1) for each of the 6 k-mer types.
4) Sum the results from step 3 to obtain the final value.

We calculate the diagonals of the p-spectra by summing the squares of corresponding row entries within the mapping function matrix for both sequences s and t, using the equation:

$$K_p(z, z) = \sum_{u \in_p} (\Phi_u^p(z))^2$$

In this equation, z represents either s or t, and we apply this calculation to both sequences to determine the diagonals for each.

For our example, we calculate this in Python using the following steps:

1) For the wild-type sequence (s), we square the row entry for each of the 6 k-mers, and then sum the result. This results in a count of 4 as each row entry is 1.
2) For the mutant sequence (t), we repeat the above process. This also results in a count of 4.

The resulting p-spectrum kernel matrix is:

Table 5: p-spectrum kernel matrix

**K**	**ATCGT (s)**	**ATAGT (t)**
**ATCGT (s)**	4	2
**ATAGT (t)**	2	4

For a more comprehensive explanation and detailed Python implementation, please see the 'get_kernel.py' script in our DrivR-Base GitHub Repository.

FG7: Amino Acid Substitution Matrices

Background: Amino acid substitution matrices play a crucial role in understanding the evolutionary dynamics of protein sequences. These matrices quantify the rates at which amino acid residues are substituted for one another during evolution. They provide valuable information about the likelihood of specific amino acid substitutions occurring in proteins. Amino acids that have a low probability of being substituted are typically those that differ significantly in their chemical composition, structure, or function. These substitutions are less common due to the functional and structural constraints imposed on protein sequences. Therefore, when a variant leads to an amino acid substitution that is associated with a low substitution probability according to the amino acid substitution matrix, it suggests a substantial change in the chemical composition of the protein. Such substitutions may have a greater likelihood of impacting the protein's function, stability, or interaction with other molecules.
By considering amino acid substitution matrices, we can gain insights into the potential functional consequences of amino acid changes caused by variants. For variants in coding regions of the genome, this information is valuable for predicting pathogenicity and understanding their

impacts on protein structure and function.

Data source: https://rdrr.io/cran/bios2mds/man/sub.mat.html

Script location: https://github.com/amyfrancis97/DrivR-Base/tree/main/FG7_aa_substitution_matrices

Extraction: To incorporate the amino acid substitution information, we use predicted wild-type and mutant amino acids obtained from the Variant Effect Predictor (VEP) output. We compared these amino acids against a collection of eight widely used amino acid substitution matrices, which were sourced from the 'sub.mat' data in the 'bio2mds' R package. Table 6 shows the substitution matrices used and their sources.

Table 6: Amino acid substitution matrices and their sources

Substitution Matrix	Source
PAM40	(Pelé et al., 2012)
PAM160	(Pelé et al., 2012)
PAM250	(Pelé et al., 2012)
BLOSUM30	(Henikoff and Henikoff, 1992)
BLOSUM45	(Henikoff and Henikoff, 1992)
BLOSUM62	(Henikoff and Henikoff, 1992)
GONNET	(Gonnet et al., 1992)
JTT	(Jones et al., 1992)
JTT_TM	(Jones et al., 1994)
PHAT	(Ng et al., 2000)

FG8: Amino Acid Properties

Background: To capture structural information and functional impacts of amino acid changes, we incorporate properties for the 20 naturally occurring amino acids using the 'AAindex' list from the 'AAsea' package in R (Reddy, 2019). The 'AAindex' list is a comprehensive collection of 536 amino acid properties compiled from various reliable sources and contains a range of important features, such as *Cruciani* properties (principal components representing amino acid interactions with chemical groups), physical properties (including hydrophobicity, helix bends, and side chain size), and chemical properties (including pKa values and chemical shifts).
Hydrophobicity, for example, plays a role in protein folding and stability, impacting the organisation and packaging of protein structures. The Cruciani properties capture the interactions between amino acids and specific chemical groups, providing information about their chemical behaviour and potential functional significance. The physical properties, including helix bends and side chain size, contribute to the three-dimensional structure and function of proteins. Additionally, pKa values provide insights into the ionization behaviour and acid-base properties of amino acids.
By incorporating these amino acid properties as encoders for the impacted amino acids in each variant, we aim to capture functionally significant physical and chemical properties between wild-type and mutant amino acids.

Data source: https://github.com/cran/aaSEA/tree/master/data

Script location: https://github.com/amyfrancis97/DrivR-Base/tree/main/FG8_aa_properties

Extraction: We use the predicted amino acid output from our FG2_vep feature group as input for these scripts. To execute these scripts, we must first ensure that the 'AAindex.rda' R data file is downloaded and that the 'config.R' file is updated with the location in which this data file has been stored. We have provided the R data file in the '/DrivR-Base/FG8_aa_properties' sub-folder. We load this dataset into our R environment and query the values for each of the 536 properties, repeating this step for both the wild-type and mutant amino acids. We then concatenate these two dataframes by column. The resulting file contains the values for each of the wild-type and mutant amino acids for every variant predicted to be coding.

FG9: ENCODE Regulatory Features

Background: The ENCODE consortium is a comprehensive database that provides valuable information on regulatory regions within the DNA (Dunham et al., 2012). Through various experimental approaches, such as investigating open chromatin states, histone enrichment, transcription factor binding, gene expression, RNA binding sites, and methylation, ENCODE offers a wealth of data that can shed light on the functional activity of genomic sites.
While these functionally related elements can play roles in coding regions of the DNA, such as the enrichment of acetylated histones, their significance is often more pronounced in non-coding regions. These functional elements play crucial roles in the regulation of transcription and gene expression. Open chromatin regions, for instance, have been found to interact with regulatory elements, while transcription factors bind to promoters or enhancers to tightly regulate gene expression (Hu and Tee, 2017). As a result, variants located within regions enriched with these regulatory elements have a higher likelihood of having functional impacts.
ENCODE serves as a valuable resource for identifying and understanding these functional elements, providing insights into the regulatory mechanisms that govern gene expression and transcriptional regulation. By leveraging the data available in ENCODE, we can gain a deeper understanding of the functional significance of genomic variants and their potential impact on gene regulation and cellular processes.

Data source: https://www.encodeproject.org/

Script location: https://github.com/amyfrancis97/DrivR-Base/tree/main/FG9_encode

Extraction: First, we download all available ENCODE files for eight different features (shown in Table 7) and convert them from bigBed to BED format.

Table 7: ENCODE Features

**ENCODE Features**	
TF ChIP-seq	Histone ChIP-seq
DNase-seq	Mint-ChIP-seq
ATAC-seq	eCLIP
ChIA-PET	GM DNase-seq

Subsequently, we concatenate all the separate files into a single file per feature group, from which we can later extract features by querying variants. We have provided the 'downloadEncode.py' script in the 'DrivR-Base/FG9_encode' directory and have also included the downloaded datasets as zipped files in the supplementary data. Please note that this script downloads all ENCODE data locally, requiring approximately 160GB of disk space.
We then extract signal values, p-values, q-values, and peaks for each unique biosample and target. For instance, Transcription Factor ChIP-Seq involves multiple transcription factor targets (e.g., CTCF) measured in various tissues (e.g., BLaER1). In cases where there are multiple values for a given biosample and target, such as replicate assays in a specific tissue, we calculate and record the mean, minimum, maximum, and range. The resulting file contains the variant location, followed by individual values for each unique biosample and target (e.g., CTCF_BLaER1_mean).

FG10: Alpha Fold Structural Features

Background: Alphafold and PDB, used together, have enabled the prediction of a vast number of protein structures (Jumper et al., 2021; Berman et al., 2000). Once we identify the protein and the specific position at which a non-synonymous coding variant occurs, we can utilize this data to enhance our understanding of the chemical and physical composition of the surrounding protein region. In this context, we extract five atomic features and two structural conformation features from the AlphaFold crystallographic information files (CIF; *.cif*). Specifically, we retrieve the x, y, and z coordinates, which are defined with respect to a set of orthogonal Cartesian axes. This description pertains to the atomic coordinates found in crystallographic structural data and is presented in Table 8.

Data source: https://alphafold.ebi.ac.uk/

Script location: https://github.com/amyfrancis97/DrivR-Base/tree/main/FG10_alpha_fold

Extraction: We use the full VEP query output from our FG2_vep feature group as input for these scripts ('file_variant_effect_output_all.txt'). The 'get_alpha_fold.py' script extracts the predicted gene names and predicted protein positions from the VEP file and converts the gene names to UniProtKB IDs. We then use the 'requests' library to fetch the appropriate file for a given variant and read it into Python. Next, we extract the relevant values for each of the structural and atom-site features at the specified protein positions. For the structural conformation features, we convert the output into a one-hot-encoded table, where a '1' in the conformation type column represents the presence of that type.

Table 8: Alpha Fold Features

Feature	Label	Description
X Atom-Site Coordinate	_atom_site.Cartn_x	The x atom-site coordinate in angstroms specified according to a set of orthogonal Cartesian axes related to the cell axes
Y Atom-Site Coordinate	_atom_site.Cartn_y	The y atom-site coordinate in angstroms specified according to a set of orthogonal Cartesian axes related to the cell axes
Z Atom-Site Coordinate	_atom_site.Cartn_z	The z atom-site coordinate in angstroms specified according to a set of orthogonal Cartesian axes related to the cell axes
Atom-Site Occupancy	_atom_site.occupancy	The fraction of the atom type present at this site
Isotropic Displacement Parameter	_atom_site.B_iso_or_equiv	Isotropic atomic displacement parameter, or equivalent isotropic atomic displacement parameter, Beq, calculated from the anisotropic displacement parameters
Structural Conformation Type	_struct_conf.conf_type_id	The type of structural conformation (e.g., BEND)
Structural Conformation ID	_struc_conf.id	The ID of the structural conformation (e.g., BEND5)

Source: The feature descriptions presented in this table have been taken or adapted from the PDBx/mmCIF dictionary (https://mmcif.wwpdb.org/pdbx-mmcif-home-page.html).

References

Berman, H. M., Westbrook, J., Feng, Z., Gilliland, G., Bhat, T. N., Weissig, H., Shindyalov, I. N., and Bourne, P. E. (2000). The protein data bank. *Nucleic Acids Research*, 28:235.

Campbell, C. and Ying, Y. (2011). *Learning with Support Vector Machines.* Morgan Claypool Publishers.

Chiu, T. P., Comoglio, F., Zhou, T., Yang, L., Paro, R., and Rohs, R. (2016). Dnashaper: an r/bioconductor package for dna shape prediction and feature encoding. *Bioinformatics*, 32:1211.

Chiu, T. P., Rao, S., Mann, R. S., Honig, B., and Rohs, R. (2017). Genome-wide prediction of minor-groove electrostatic potential enables biophysical modeling of protein–dna binding. *Nucleic Acids Research*, 45:12565–12576.

Dunham, I., Kundaje, A., Aldred, S. F., Collins, P. J., Davis, C. A., and et al., F. D. (2012). An integrated encyclopedia of dna elements in the human genome. *Nature 2012 489:7414*, 489:57–74.

Friedel, M., Nikolajewa, S., Sühnel, J., and Wilhelm, T. (2009). Diprodb: a database for dinucleotide properties. *Nucleic Acids Research*, 37:D37.

Gonnet, G. H., Cohen, M. A., and Benner, S. A. (1992). Exhaustive matching of the entire protein sequence database. *Science (New York, N.Y.)*, 256:1443–1445.

Henikoff, S. and Henikoff, J. G. (1992). Amino acid substitution matrices from protein blocks. *Proceedings of the National Academy of Sciences of the United States of America*, 89:10915–10919.

Hu, Z. and Tee, W. W. (2017). Enhancers and chromatin structures: regulatory hubs in gene expression and diseases. *Bioscience Reports*, 37:20160183.

Jones, D. T., Taylor, W. R., and Thornton, J. M. (1992). The rapid generation of mutation data matrices from protein sequences. *Computer applications in the biosciences : CABIOS*, 8:275–282.

Jones, D. T., Taylor, W. R., and Thornton, J. M. (1994). A mutation data matrix for transmembrane proteins. *FEBS letters*, 339:269–275.

Jones, S., Shanahan, H. P., Berman, H. M., and Thornton, J. M. (2003). Using electrostatic potentials to predict dna-binding sites on dna-binding proteins. *Nucleic Acids Research*, 31:7189.

Jumper, J., Evans, R., Pritzel, A., Green, T., Figurnov, M., Ronneberger, O., Tunyasuvunakool, K., and et al., R. B. (2021). Highly accurate protein structure prediction with alphafold. *Nature 2021 596:7873*, 596:583–589.

Karimzadeh, M., Ernst, C., Kundaje, A., and Hoffman, M. M. (2018). Umap and bismap: quantifying genome and methylome mappability. *Nucleic Acids Research*, 46:e120–e120.

McLaren, W., Gil, L., Hunt, S. E., Riat, H. S., Ritchie, G. R., Thormann, A., Flicek, P., and Cunningham, F. (2016). The ensembl variant effect predictor. *Genome Biology*, 17:1–14.

Ng, P. C., Henikoff, J. G., and Henikoff, S. (2000). Phat: a transmembrane-specific substitution matrix. predicted hydrophobic and transmembrane. *Bioinformatics (Oxford, England)*, 16:760–766.

Pelé, J., Bécu, J. M., Abdi, H., and Chabbert, M. (2012). Bios2mds: An r package for comparing orthologous protein families by metric multidimensional scaling. *BMC Bioinformatics*, 13:1–7.

Pollard, K. S., Hubisz, M. J., Rosenbloom, K. R., and Siepel, A. (2009). Detection of non-neutral substitution rates on mammalian phylogenies. *Genome Research*, 20:gr.097857.109.

Reddy, R. (2019). aasea: Amino acid substitution effect analyser version 1.1.0 from cran.

Rohs, R., West, S. M., Sosinsky, A., Liu, P., Mann, R. S., and Honig, B. (2009). The role of dna shape in protein-dna recognition. *Nature*, 461:1248.

Rozenberg, J. M., Shlyakhtenko, A., Glass, K., Rishi, V., Myakishev, M. V., FitzGerald, P. C., and Vinson, C. (2008). All and only cpg containing sequences are enriched in promoters abundantly bound by rna polymerase ii in multiple tissues. *BMC Genomics*, 9:1–13.

Sayers, E. W., Bolton, E. E., Brister, J. R., Canese, K., Chan, J., Comeau, D. C., Connor, R., Funk, K., Kelly, C., Kim, S., Madej, T., Marchler-Bauer, A., Lanczycki, C., Lathrop, S., Lu, Z., Thibaud-Nissen, F., Murphy, T., Phan, L., Skripchenko, Y., Tse, T., Wang, J., Williams, R., Trawick, B. W., Pruitt, K. D., and Sherry, S. T. (2022). Database resources of the national center for biotechnology information. *Nucleic acids research*, 50:D20–D26.

Siepel, A., Bejerano, G., Pedersen, J. S., Hinrichs, A. S., Hou, M., Rosenbloom, K., Clawson, H., Spieth, J., Hillier, L. D. W., Richards, S., Weinstock, G. M., Wilson, R. K., Gibbs, R. A., Kent, W. J., Miller, W., and Haussler, D. (2005). Evolutionarily conserved elements in vertebrate, insect, worm, and yeast genomes. *Genome Research*, 15:1034–1050.

Vinogradov, A. E. (2003). Dna helix: the importance of being gc-rich. *Nucleic Acids Research*, 31:1838–1844.

Woodruff, D. S. (2001). Populations, species, and conservation genetics. *Encyclopedia of Biodiversity*, page 811.
